# The effect of the tongue in groove technique on the nasolabial angle and nasal tip projection

**DOI:** 10.1186/s40902-020-00261-8

**Published:** 2020-06-08

**Authors:** Fatemeh Khabir, Mehdi Sezavar, Behnam Bohluli, Vahid Mesgarzadeh, Hamidreza Tavakoli

**Affiliations:** 1 Department of Oral and Maxillofacial Surgery, Bu Ali Hospital, Islamic Azad University, Tehran, Iran; 2 Department of Oral and Maxillofacial Surgery, Craniomaxillofacial Research Center, Bu Ali Hospital, Islamic Azad University, No. 17, Golestan Alley, Shahrak Gharb Ave, Tehran, Iran; 3 Department of Oral and Maxillofacial Surgery, University of Toronto, Toronto, Canada

**Keywords:** Rhinoplasty, Tongue in groove technique (TIG), Nasal tip projection, nasolabial angle

## Abstract

**Background:**

The tongue in groove technique (TIG) is a useful technique for the correction of the nasal tip projection and the nasolabial angle. The purpose of this study was to determine the utility of this technique for nasal tip rotation and projection correction in the Iranian society.

**Methods:**

This is a retrospective clinical trial study of 20 patients undergoing open septo-rhinoplasty using TIG technique from January 2017 to August 2019 at the oral and maxillofacial unit of Bu Ali Hospital and private sector. Preoperative and postoperative profile view photographs were compared to assess the changes in tip projection and rotation.

**Results:**

Fifteen patients (75%) had normal angular size, and 5 of them (25%) were not within the normal range after the surgery. The Fisher exact test showed that this success was statistically significant (*P* = 0.006). Ten patients (50%) had normal projection size, postoperatively. The Fisher exact test showed that this effect was statistically significant (*P* < 0.01)

**Conclusion:**

The study demonstrated the benefit of TIG on the correction of nasal tip projection and rotation.

## Background

One of the most challenging surgeries in the field of aesthetic is rhinoplasty [1, 2], among many useful methods providing desired results, tongue in groove (TIG) is a strong suture technique allowing conservative surgery to manage tip projection and rotation [3]. This method has been performed to correct the caudal septum deviation, as well as excessive columellar show [4]. It can be used in either open or closed rhinoplasty.

**Fig. 1:**
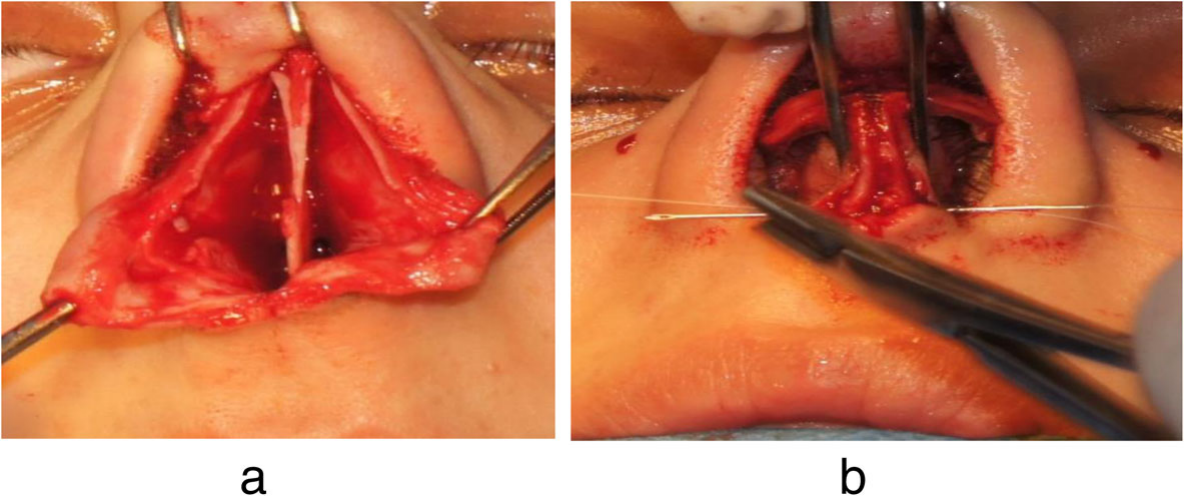
Surgical procedure. **a** It shows a membranous septum detachment, exposing the septum between the medial crura. **b** It refers to perform suturing of the medial crura and the septum together

The axiom in this approach is adopting and relocating the medial crura in its right, stable position before suturing is performed [2, 5]. The extension of cartilaginous septum will be placed in a groove surgically created between the medial crura (Fig. 1). The suture position has a very important role in rotation and projection of the nasal tip [2]. A more superior position of the suture along the vertical axis of the septum results in projection and rotation increase. Also, the movement of the suture along the horizontal axis of the septum will cause increase in nasal tip rotation with no nasal projection changes.

Although the literature show the effectiveness of this technique in European and American human studies [1, 6], we failed to find an article assessing the efficacy of this method in the Iranian society; so the aim of this study was to confirm this effectiveness through a clinical study.

## Methods

Through the present study of a consecutive series of patients, undergoing primary septo-rhinoplasty in Bu Ali Hospital and private clinics from January 2017 to August 2019, we performed the TIG technique on 20 patients having tip deformities in their preoperative documents. Ethical approval was given in May 27, 2018, by the research ethics committee of Islamic Azad University, Tehran Branch (approval ID is IR.IAU.DENTAL.REC.1397.022). Hiring this ethical approval and based on the consent form for each patient, we performed this study.

We analyzed photographs of 2 men and 18 women aged between 18 and 55. Exclusion criteria were as follows: the history of previous septo-rhinoplastic surgery, trauma to the nose, normal range of nasolabial angle (90–110°), and normal tip projection according to the Goode method assessment (55–60%) [3]. Also, any jaw deformity requiring orthognathic surgery and any craniomaxillofacial syndrome causing nose deformation were excluded.

**Fig. 2:**
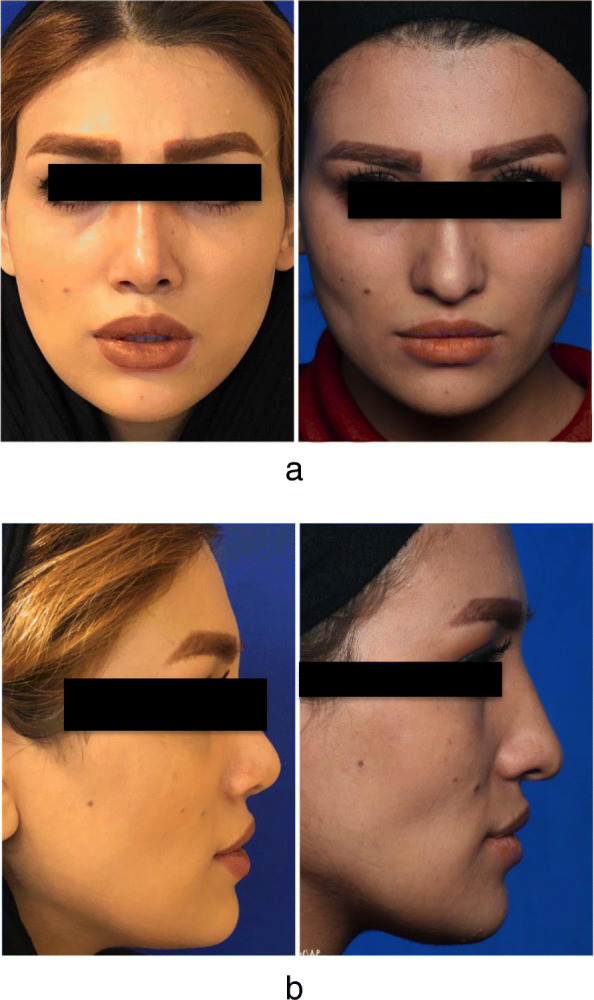
Clinical presentation before and after surgery. **a** Frontal view before and after surgery. **b** Lateral view before and after surgery

Profile view photographs were taken pre- and post-surgery according to the standardized clinical photography (1:1) (Fig. 2). Evaluation of the nasolabial angle by using the Golden Rhino Software and the Goode method for measuring nose projection was performed. All patients were followed up at least for 6 months. Nasolabial angle and nasal tip projection changes were judged by statistical testing named the Fisher exact test.

## Result

The study was designed to assess the effect of TIG on nasolabial angle and tip projection changes in the Iranian society. Twenty eligible patients including 18 women and 2 men aged between 18 and 55 underwent open septo-rhinoplasty surgery. None of the patients had a history of rhinoplasty, nasal trauma, and jaw and craniomaxillofacial abnormalities.

The distribution of nasolabial angle in patients before and after TIG surgery show that before surgery, all patients had a nasolabial angle size out of the normal range (Table 1). In the postoperative period, 15 patients (75%) had normal angular size. The Fisher exact test showed that this success was statistically significant after treatment (*P* = 0.006).

**Table 1:** Distribution of patients undergoing rhinoplasty in terms of nasolabial angle before and after surgery

	Normal range	Out of normal range	*P* value
Before surgery	0	20	0.006
After surgery	15	5	

Pre- and postoperative results of nasal tip projection and their comparison showed that prior to surgery, none of the patients’ tip position were within the normal range. Postoperatively, 10 patients (50%) had normal projection size (Table 2). The Fisher exact test showed that this effect was statistically significant (*P* < 0.01).

**Table 2:** Distribution of patients undergoing rhinoplasty in terms of nasal tip projection before and after surgery

	Normal range	Out of normal range	*P* value
Before surgery	0	20	< 0.01
After surgery	10	10	

## Discussion

Despite technical advances, rhinoplasty has progressed with an inclination toward minimally invasive surgical techniques. TIG technique is one of the most successful procedures and also a non-aggressive one. This method is first described by Kridel et al. [7] in 1999 and continued by Shah and Miller [8], then followed by Toriumi [2] and Guyuron and Brow [3]. The positive effect of TIG is determined in tip deformities and septal deviation of the lower third part of the nose and columellar show, which had a remarkable and excellent functional and aesthetic outcome and long-lasting support [7].

In contrast to previously described techniques, TIG does not rely on graft harvesting for placement between the medial crura, yet even can play its role in tip projection and nasolabial angle rotation as well as septal deviation successfully [9, 10]. In addition to the mentioned advantages, tip rotation can perform more controlled than routine maneuvers, based on unexpected contracture to gain a proper tip [6, 11]. Patients did not complain any abnormal rigid sensation in the subnasal because of the columellar graft application. Caudal septal deviation also can be corrected without any excision in the septum which may lead to droopy nose deformity [12, 13].

Recently, Lohuis and Datema [6] published a description of TIG advantages in revision rhinoplasty which can be performed in combination with graft augmentation. As a result, TIG is a valuable multi-purpose technique which can be used for correction of excessive columellar show, short nose, droopy nose, reformation of septal deviation, tip plasty in association to improving nasolabial angle, and tip projection correction [5, 14].

In our study, which is the first published data describing the effectiveness of this method, advantages of TIG in the correction of nasolabial angle and nasal tip projection in the Iranian society had its reproducibility as well as predictability (Tables 1 and 2). The most critical advantage of this method is that we can still choose a minimally invasive technique such as TIG suturing which may be laid in modern rhinoplastic techniques.

## Conclusion

Finally, TIG is a predictable technique and has high success rate which can adjust the medial crura in a proper position in respect to the septum as a tongue. It can be recognized as a long-lasting stable method with appropriate functional and aesthetic results.

## Data Availability

Data in the Iranian society was not available.
